# Perspectives of on‐farm biosecurity and disease prevention among selected pig veterinarians and pig farmers in Sweden

**DOI:** 10.1002/vro2.68

**Published:** 2023-07-18

**Authors:** Hedvig Gröndal, Krista Tuominen, Susanna Sternberg Lewerin

**Affiliations:** ^1^ Department of Biomedical Sciences and Veterinary Public Health Swedish University of Agricultural Sciences (SLU) Uppsala Sweden

## Abstract

**Background:**

Biosecurity is important in preventing the spread of infectious diseases in animal production. Previous studies have identified a disparity between the biosecurity recommendations provided by veterinarians and the actual practices implemented by farmers. This study compared group discussions with a few key actors among Swedish pig veterinarians and farmers on pig farm biosecurity.

**Methods:**

Two focus group discussions were conducted, one with five Swedish pig veterinarians and one with three pig farmers, to explore their views on pig farm biosecurity and efficient biosecurity measures. The discussions were analysed to identify differences and similarities in how biosecurity was perceived.

**Results:**

The study identified differences between the veterinarians and pig farmers in how they perceived good biosecurity and the level of biosecurity in Swedish pig herds. The veterinarians perceived that adhering strictly to the farming system and its barriers was essential for good biosecurity. The biosecurity in the pig farms was often considered inadequate. The veterinarians described difficulties in biosecurity‐related communication with the farmers. The pig farmers valued the flexibility of the farming system over strict barriers and described that the level of biosecurity was good in Swedish pig herds. However, both groups also shared similar views regarding the challenges in farm biosecurity. They highlighted that biosecurity measures with proven efficacy are important for farmer motivation.

**Conclusions:**

This limited study suggested that different perspectives on biosecurity can contribute to communication difficulties between pig farmers and veterinarians. Acknowledging both the differences and similarities of the different perspectives may help improve cooperation and communication in biosecurity‐related questions.

## INTRODUCTION

Biosecurity plays an important role in preventing infectious animal diseases. Voluntary programmes, as well as legal requirements for biosecurity plans on animal holdings, are becoming increasingly common.^1^, ^2^ In pig production, many diseases can be prevented and controlled by internal and external biosecurity measures.^3^ Although these measures are biologically well‐founded,^4^ the value of each individual measure is difficult to assess and risk assessment models have been developed to address this challenge.^5^, ^6^ These models confirm that combinations of measures are required to reduce the risk of introduction and spread of infections within pig farms. The variability in risk for different diseases, different farms and different transmission routes presents a challenge in itself when it comes to motivating individual farmers to implement preventive measures.^4^ The implementation is further affected by several factors, such as the farmer's personality, gender, age, education level and access to information.^7^, ^8^, ^9^ Successful disease prevention is difficult to measure, as its result is the absence of an event that might, or might not have occurred without the preventive effort. Hence, motivating biosecurity routines is challenging. Several studies indicate that the implementation of on‐farm biosecurity measures is often inadequate.^10^, ^11^, ^12^ Different drivers and priorities have been noted in farmers’ perspectives of biosecurity,^13^, ^14^ and that of veterinarians and farm advisors.^15^

Social scientists have argued for a multifaceted understanding of farmers’ biosecurity practices, which take into account the farmers’ local knowledge.^16^, ^17^, ^18^ Although farmers tend to be very concerned about diseases, their practices do not always follow veterinary advice.^19^ One aspect of this pertains to conflicting ideals of ‘good farming’. One study^20^ reported how the ideals of the traditional and independent stockkeeper tend to be in conflict with the ideals of the large commercial farmer, who in turn tend to align more with the veterinarians’ ideals of farm biosecurity. Another study^18^ argued that an understanding of farmers’ biosecurity practices as inadequate is reductionist since it does not account for how traditions are combined with the implementation of official recommendations. Veterinary advice is only one of many elements that farmers take into account in their work. One study concluded ‘It is not the case that farmers operate in a “knowledge vacuum” that vets attempt to fill’.^17^ In the farmers’ everyday practice, veterinary advice is combined with other sources of knowledge, their own values and what is actually doable on the farm. Moreover, several authors have argued that what is ‘doable’ is to recognise and, to some extent, accept the existence of biosecurity threats.^21^, ^22^

Previous studies have reported that discrepancies between veterinary biosecurity recommendations and farmers’ practices are common, which might create tensions in the relationship between these actors. To explore this, the current study compared group discussions with some key actors among Swedish pig veterinarians and pig farmers faced with similar challenges relating to pig health.

## METHODS

Two focus group discussions were organised with key actors among Swedish pig veterinarians and Swedish pig farmers.^23^ The original purpose was to elicit information about feasible interventions against livestock‐associated meticillin‐resistant *Staphylococcus aureus* (LA‐MRSA), as a basis for selecting interventions to test in a model of this zoonotic pathogen. Therefore, we chose to talk to key actors with substantial knowledge of Swedish pig production. The veterinarians were selected by direct invitation to pig veterinarians working with the veterinary advisory services Farm and Animal Health. This is the main advisory organisation for pig farmers, covering the majority of all commercial pig farms in Sweden. Their veterinarians have a strong contact network among Swedish pig veterinarians and a large impact on their field practices. The organisation runs a national biosecurity scheme, in addition to several disease‐specific control programmes. The farmers were selected by inviting the entire board of the Swedish Pig Farmers Association, that is, the national representatives of this farmer group. The final selection of participants depended on who could participate on the date and time that suited most of them, resulting in five veterinarians (two female, three male) and three pig farmers (one female, two male). All veterinarians had extensive experience working with pig health and participated in the development and implementation of national pig health programmes. The farmers were all large pig producers with farrow‐to‐finish herds, with long‐standing knowledge of Swedish pig production in general, that were used to represent their profession in different discussions at the national level. The participants lived in different parts of Sweden in the areas where most pig farms are located.

Both meetings were conducted via Zoom (Zoom Video Communications, Inc.) on two consecutive days. The meetings were facilitated by the first author, based on a predetermined discussion guide (Supporting Information S1). Before the start of the discussion, all participants were informed about the purpose of the discussion, that the meeting would be recorded, that they would remain anonymous in all publications and presentations of the results and finally that they could withdraw from the study at any time. The recording was only started after consent had been given by all participants.

As the aim was to understand what interventions would be feasible in pig farms, the discussions were initiated around this subject, specifically mentioning LA‐MRSA and the possibility of its detection in Swedish pig herds. Participants were given a few examples of potential interventions to stop the within‐herd spread and asked to think of other means to achieve this and discuss the feasibility of different measures in Swedish pig herds.

The recorded discussions were transcribed and the transcripts were analysed manually. First, the transcripts were read in full and empirical codes were created. As the codes differed between veterinarians and farmers and constituted two different ways of describing good biosecurity, we ordered data according to these two approaches, conceptualised as: *Staying true to the system* and *Flexibility*. In addition, the detailed issues that were described similarly in the two groups were coded and organised into the following themes: *Developments in the pig industry*, *Motivating actions* and *Individual drivers*.

## RESULTS

### Different perspectives on biosecurity

#### Veterinarians—stay true to the system

In the veterinarians’ discussion, the initial narrative of Swedish pig farming was characterised by several shortcomings in biosecurity. The veterinarians described it as difficult to communicate with farmers about these shortcomings and the pig farmers did not understand them. For example, one veterinarian described a feeling of speaking a completely different language. A recurrent theme in the veterinarians’ descriptions was that biosecurity was challenged by farmers’ tendency to not stay true to the ‘system’. With the term ‘system’, the veterinarians referred both to how pigs were organised into different age groups that are kept separate and how production was carried out in a batchwise ‘all in, all out’ principle. One veterinarian stated and the others agreed:

‘This, upholding a batchwise and sectioned breeding, that is what I see that they are sloppy with everywhere today’.

The veterinarians described the system‐breaking practices as occurring on all kinds of pig farms. Even on the farms described as ‘aware’ and the ‘best’ in relation to biosecurity, the system was continuously challenged:

‘It was great, it was like super, they are excellent. They have this fantastic production and very good biosecurity in many ways. It's just that they still move pigs around’.

Throughout the discussion, the veterinarians described ‘the system’ as a set of rigid rules that should never be broken. One veterinarian said:

‘When you choose a system then you can't just say that I want other rules than the system requires … You can't have stragglers all the time that have to be moved backwards in the system. You can't have one sow farrowing at the wrong time … I think this applies to many infectious diseases, we manage them by having a very controlled production. So the foundation, or part of it, is to really control the production and not accept any exemptions’.

For the veterinarians, the primary function of the system was to stop the spread of disease:

‘I also think that we have the system with sectioned, batchwise, production to streamline, but most of all to keep diseases at bay’.

A common breach of the system, as described by the veterinarians, was to assemble pigs that had not reached the expected slaughter weight in a ‘buffer section’. By this, some farmers created a separate system that opposed the original system. As the quote below indicates, these buffer sections were described as extremely problematic for biosecurity:

‘We reduced the respiratory problems in a holding, where we've sampled a lot and now it was negative practically all the way to the fattening units. But they also have buffer units and it was like a bomb, all of it’.

The veterinarians brought up another example of challenging the system, bringing in nursing sows when the litters were too large. Staying true to the system would mean that the piglets that could not be fed by their biological mother would instead be euthanased; the pigs that did not gain weight like the others would be culled or placed in a separate production line.

‘I'm thinking that one intervention that would be very effective is to euthanase all that deviate from the norm … at every stage. … Generally, it's a very economic attitude’.

Staying true to the system was central for veterinarians, which was evident when they discussed vaccinations. While vaccinations reduce the risk of disease, they can be problematic because they might encourage farmers to ignore the separation of animals.

‘More vaccines, that's both an advantage and a disadvantage: “A little mycoplasma, we no longer need to keep sectioning because of that, we have good vaccines. Or Lawsonia, we'll vaccinate so it goes away”’.

The veterinarians also described that farmers not only challenged the system by moving pigs but also that the staff tended to ignore hygiene routines and thereby spread pathogens between different stables. The veterinarians described such routines as easy to follow; thus, the non‐compliance was perceived as irrational.

‘I have a good example where we had problems with respiratory infections in the growing units, and we introduced a total change of clothing when they entered and the respiratory problems basically vanished … Quite simple measure, really. Some boots hanging outside, pants that they put on, easy … You don't have one employee per section … it can be practically an autostrada back and forth. They go and help each other and whatever between sections and that will not turn out well … But what they have in place now is that they finish all the time. We've tried to find simple things’.

Moreover, the veterinarians described how farmers tended to implement hygiene routines after a disease outbreak had occurred when it, according to the veterinarians, was too late.

‘Making them understand the internal biosecurity … it's very, very, unusual, I feel, that they change clothes and boots between different age groups, and it almost takes an outbreak … to make it happen. Even though we know it would be desirable’.

The veterinarians also mentioned their different roles:

‘We're both inspector and advisor and it's about the balance. But there is still a difference to when a salesperson comes and tells you things and when it's actually your own vet’.

The veterinarians felt alone in prioritising biosecurity and disease prevention, while other actors were focusing on production output and short‐term financial gains.

‘It's our own agricultural experts, in our own organisation, they don't understand us, we don't speak a common language’.

#### Farmers: flexibility of the system, safeguarding other values

In the discussion with pig producers, a different perspective on biosecurity emerged. Biosecurity was described as generally good in Swedish pig production:

‘I think … that the awareness among us pig producers about the benefits of good biosecurity, it's probably, like, very high’.

The producers described prevention of disease spread as always present in their own and their colleagues’ minds:

‘But you always carry the thought with you: How can I avoid transmission of the infection?’

The producers described that practices of mixing animals from different sections and groups existed on their farms. However, this was expressed in very different terms compared to the veterinarians: first, the producers described themselves as well aware of the disease risks.

‘But everything must also work practically. Everything must run smoothly, so one does as well as one can … you never move pigs that have a problem. You mustn't move pigs backwards in the system’.

Second, they emphasised how disease control sometimes conflicted with other values. They stated that it was sometimes necessary to mix pigs despite the risks. A recurring perspective in the producers’ discussion was their other priorities in addition to disease control; consequently, they sometimes made conscious deviations from biosecurity routines.

One example was when a producer described balancing the risk of disease against the gains from buying animals.

‘We chose the risk of getting something contagious against the benefit of upgrading our breeding stock … after discussing back and forth we arrived at, if we implement quarantine and choose holdings with a good health status the risks should be relatively low, but we'll see if this was right’.

Importantly, as the quotation below illustrates, the disease control might, according to the producers, conflict with the ‘optimal system for the pigs’.

‘Sometimes it may be that … biosecurity is in conflict with the optimal system for the pigs … If you have a gilt that is a little thin and has large piglets then you may want to wean them a week early and mix the piglets with another group to, well, make it as good as possible for the animals … we do those things every now and then but we're aware of that it isn't good for the internal biosecurity. But that's a balance you must make’.

Like the veterinarians, the producers described that sometimes a disease outbreak was needed to follow hygiene routines strictly. However, in contrast with the veterinarians, the producers did not describe this as a flawed form of practice, but as natural:

‘Of course, the more infections that circulate the more you care, it's quite natural. Then, it's like … maybe you don't care as long as everything works and you don't have any major problems. But perhaps … the details maybe you address first when you have the knife against your throat and there is a problem’.

Moreover, the farmers described that following basic hygiene routines was not always easy. For example, when disease prevention conflicted with the staff's social need to have breaks together:

‘Well we have top status but it's not always easy to comply with all the time in practice … it's mainly the external biosecurity that I find difficult, for example, the staff wouldn't be allowed to have coffee together … without changing clothes, and it feels like the risks of getting a disease may be larger elsewhere’.

### Topics of concern for both groups

#### Developments in the pig industry

Although the discussions in the two groups were characterised by differences, we also identified similarities. Both groups highlighted challenges related to steadily increasing farm sizes and genetic advances leading to larger litters. However, while the veterinarians worried about shorter farrowing intervals jeopardising the fundamental idea of the batchwise system, the farmers mentioned the opportunities to mix batches and create better welfare for the sows.

As mentioned above, the veterinarians presented the genetic developments leading to larger litter sizes as a challenge to the system because it created a need for nursing sows. The farmers also mentioned this, however, they primarily described it as an issue related to older buildings. They discussed possible solutions in new systems, with larger pens and milking cups for supplementary feeding. Both groups expressed concerns about genetic development leading to higher animal density.

Veterinarian: ‘It gets full, full, full, they have growing pens built for ten pigs but now there are fifteen … more pigs that are all nice and even and grow at the same rate’.

Farmer: ‘If you look at the production the pigs are 10 kg heavier than 15 years back in time. The slaughter weight has increased … so that you have almost one more pig in the pens than what you did back then’.

Both farmers and veterinarians also mentioned the challenges of trying to expand and increase herd size with old buildings, as compared to building new housing with larger pens.

#### Motivating actions

Both groups highlighted the importance of being able to show the positive effects of biosecurity. The farmers mentioned follow‐up of indicators during veterinary herd visits, using their own data to show their staff how biosecurity breaches may be detrimental and to celebrate when good work paid off.

‘Those are things we can celebrate, when we have broken the record in number of weaned, when we had zero treated sows, like, and the numbers of lameness, and piglet diarrhoea, and treatments of diarrhoea among the growers. You follow up and see “Why was there a peak there” … just that you have an awareness and can discuss about why it is like this or that’.

The veterinarians acknowledged that it might be hard to motivate improved biosecurity when the results were not visible. They wished for a ‘litmus test’ to demonstrate how infections spread through a herd, of shared experiences of successful herd interventions with demonstrable results and redeemable problems discovered by simple follow‐up of disease data.

The veterinarians proposed that individual production indicators could be problematic, such as striving for low piglet mortality might lead to keeping runt pigs that must be managed by either mixing with subsequent batches or in a buffer pen:

‘Again, we're talking about mortality, that is, mortality up until weaning. We're talking percentages and you're bad if you have a high mortality. So, it's wrong, counterproductive goals that are set up, in my opinion’.

Paradoxically, as suggested by one of the veterinarians, the good animal health status in Sweden may reduce the incentives for biosecurity. As described above, both groups recognised a disease outbreak as one of the strongest incentives for strict biosecurity measures.

Regarding the original purpose of the meetings, which was to gain knowledge about feasible interventions to reduce LA‐MRSA, the participants found it difficult to propose any measures. However, although some challenges were mentioned, the farmers stated that almost any intervention could be implemented if a positive effect could be expected.

‘If only someone gives a clear directive, I think any farm can do it … if you know what result you can expect. Of course, if you know that you really can eradicate something by making a strong effort then I think most people could do very much’.

Despite the assurance that this was purely theoretical, the veterinarians were hesitant to suggest any interventions for which they had no experience or evidence, reflecting the guiding principles of veterinary practice.

#### Good life beyond biosecurity

While recognising the need for economic profit, both groups acknowledged the fact that biosecurity was not the only priority for the farmers. Preventing disease was described as important, but sometimes in conflict with a ‘good farmer's life’, which was also important to the farmers.

‘It's a balance. We have to live with this as well … it mustn't become … it may be secure, but it must also be nice …’

The veterinarians also, to some extent, recognised the need for the farmers to prioritise other things in life than animal disease control. As described above, this was presented mainly as a challenge, but also as an opportunity.

‘I like it in a way, this that “Yes I want a good life, that's better than lots of money” … It's us who need to find out, what are the goals on this holding? … how can I incentivise my advice?’

## DISCUSSION

In this small study, the farmers described Swedish pig herds as having good biosecurity. This statement was supported by the generally low disease prevalence and low use of antimicrobial drugs. Even so, they acknowledged that there might be room for improvement. Previous studies have reported that on a general level, Swedish farms have room for improvement in their biosecurity.^24^, ^25^ In contrast to the farmers, the veterinarians took a more negative stance on the current status of farm biosecurity, describing it as inadequate and that communicating with farmers about biosecurity was difficult.

The seemingly different views in the two groups could be due to within‐group dynamics during the discussions. It could also be related to the veterinarians perceiving biosecurity as an essential tool in animal disease control, which is one of the main interests of the veterinary profession, while for farmers, it is simply one of many aspects of good animal husbandry. The participating veterinarians were experienced animal health advisors and expected to be highly aware of biosecurity aspects in animal production.^26^ For the farmers, many other things beyond disease prevention and control, for example, to ‘have a good life’, were important and influenced their decisions. However, the diverging perceptions on the state of biosecurity and the communication challenges could be linked to veterinarians and farmers having different ways of describing good biosecurity. For the veterinarians, staying true to the system and not breaking barriers between different groups of pigs were framed as key. The farmers described flexibility as crucial, both for securing overall biosecurity and preserving other values. In line with previous studies, producers thus described a tendency to accept and ‘live with’ biosecurity threats in a way that is not recognised by official recommendations.^21^, ^22^ That farmers needed to be flexible and adaptive and that rigid rules were problematic for them, has been reported previously.^27^

Although the discussions were characterised by differences in perspectives on biosecurity and biosecurity‐related issues, we also identified similarities. For example, the veterinarians to some extent appreciated farmers’ desire to have a good life and recognised that the farmers saw the importance of healthy, happy pigs. Consequently, their discussion also highlighted the need to come up with biosecurity advice that was not too onerous and, further, that the health and wellbeing of the pigs could be used as incentives.

The farmers implied that they were willing to implement any necessary disease control measures if the measures were proven to be effective. It is likely that farmers are concerned about the time or monetary investments that interventions might require and the costs of biosecurity and disease prevention measures have been identified as barriers to implementing disease prevention measures.^8^, ^28^ However, guaranteeing the success of any control measure is impossible.

The small number of interviewed participants limits the conclusions that can be made based on the discussions and this should be regarded as a pilot investigation. We chose to include representatives from the veterinary advisors and producers who are key actors in the Swedish pig industry to elicit information from actors used to discuss biosecurity on the industry level as well as on individual farms. The original purpose was to understand what was seen as feasible on‐farm interventions in case of an infectious disease outbreak; hence, we were seeking informants with experience from both farm and national levels. The veterinarians were experienced pig veterinarians working with preventive animal health and the farmers were experienced producers and representatives of the pig producers’ organisation.

The focus of the discussions quickly turned to various aspects of biosecurity, partly guided by the facilitator but also governed by the participants, indicating that this was a topic of high interest for both parties. Despite the highlighted diversities, there was an underlying agreement about important challenges and end goals, although these were phrased differently in the respective groups. These observations may provide inspiration for future research and discussions about communication between veterinarians and farmers.

We suggest that the different perspectives on biosecurity can partly explain difficulties in communication between farmers and veterinarians, as well as the lack of implementation of official biosecurity policies in farms. However, we also argue that it is important to acknowledge not only the differences between farmers’ and veterinarians’ perspectives but also identify the similarities because these can provide a common ground for cooperation and improvement.

## AUTHOR CONTRIBUTIONS

All authors contributed to the planning and execution of this study. All authors participated in the focus group discussions, which were facilitated by Hedvig Gröndal. Susanna Sternberg Lewerin and Hedvig Gröndal analysed and interpreted the data. All authors contributed to authorship and approved the final version.

## CONFLICTS OF INTEREST STATEMENT

The authors declare they have no conflicts of interest.

## ETHICS STATEMENT

The authors confirm that the ethical policies of the journal, as noted on the journal's author guidelines page, have been adhered to. According to national legislation, no ethical approval was required. All participants were informed of the purpose and the recording prior to the discussions. Consent was recorded for all participants

## Supporting information

S1. Interview guide focus group discussions.

## Data Availability

The data that support the findings of this study are available from the corresponding author upon reasonable request.
