# Supplementary material for: Perspectives of on‐farm biosecurity and disease prevention among selected pig veterinarians and pig farmers in Sweden
Source: Vet Rec Open. 2023 Jul 18;10(2):e68. doi: 10.1002/vro2.68 (PMC10354000; doi:10.1002/vro2.68)
Supplement: Supplementary file 1 — S1. Interview guide focus group discussions. [file VRO2-10-e68-s001.pdf]

Supporting Information S1

Interview guide focus group discussions (translated from Swedish)

1. Brief personal introduction by the Principal Investigator (Susanna Sternberg Lewerin) followed by: Recap of what was written in the invitation, that this is part of a project on where we try to model within-herd spread of livestock-associated meticillin-resistant *Staphylococcus aureus* (LA-MRSA), but the model can also be used for other infections. We need your help with ideas of what can be done in a pig herd to stop and/or reduce the spread of infections. Hedvig Gröndal (HG) will lead the discussions and the PhD student in the project (Krista Tuominen, KT) and I will take notes and ask questions if there is something we don't understand, so that we capture your views correctly.

2. Brief personal introduction by PhD student (KT).

3. Brief personal introduction by focus group leader (HG) followed by:

What we want you to do is speculate freely, whatever comes into mind, about ways to manage an outbreak of infectious disease in pig herds. Some things are important. The ideas and suggestions will only be used in our theoretical model, so you don't have to consider limits such as for example costs and politics. Actually, you don't have to feel that your suggestions have to work, it's enough if you think they might work. It could be any kind of infectious disease, not necessarily LA-MRSA, or even a serious disease. Maybe you have different ideas depending on what disease it's about, and that's OK of course, you're welcome to brainstorm around that. And you may also describe things that are already used to manage disease outbreaks in pig herds. We're not really after things that are done to keep diseases out of the herds, we would like to hear your ideas about when the disease is already present in the herd. I also want to emphasise that no ideas or suggestion will be regarded as stupid, wild, or too fanciful. I'm a sociologist and it would be welcome if you can, if possible, explain so that I too understand. I still might not understand and some stupid questions from my side may come up.

I also want to clarify that we will record the discussions but the recording will we deleted after we've transcribed it and we, if we use this material in any kind of text, of course will anonymise everything you say. Now I'll let you speak freely, and you may also remain silent if you need it. If there are many people wanting to talk and you feel that you cannot make yourself heard, as video meetings can be difficult, you can wave your hand, write in the chat or simply call out and I will moderate a list of speakers. Otherwise I or any of the others may interrupt if we need to ask something or need clarification, or if we feel you stray too far away from the topic, but otherwise we will remain quite passive.

So, let's go! What are your ideas and thoughts? Large and small!

4. Additional questions:

Tell us about your experiences from stopping disease spread within pig herds. What diseases (if no suggestions come up: diarrhoea, respiratory infections)?. What did you do? What was difficult? What worked? What could be developed further?

Is there a difference between different types of infections? Which ones? What differences? Differences between serious and more common diseases?
